# Expression and pathological effects of periostin in human osteoarthritis cartilage

**DOI:** 10.1186/s12891-015-0682-3

**Published:** 2015-08-21

**Authors:** Ryota Chijimatsu, Yasuo Kunugiza, Yoshiaki Taniyama, Norimasa Nakamura, Tetsuya Tomita, Hideki Yoshikawa

**Affiliations:** 1 Department of Orthopaedic Surgery, Osaka University Graduate School of Medicine, 2-2 Yamadaoka, Suita, Osaka Japan; 2 Department of Orthopaedic Surgery, Osaka University Graduate School of Frontier Bio Science, 2-2 Yamadaoka, Suita, Osaka Japan; 3 Department of Orthopaedic Surgery, Japan Community Healthcare Organization Hoshigaoka Medical Center, 4-8-1 Hoshigaoka, Hirakata, Osaka Japan; 4 Department of Clinical Gene Therapy, Osaka University Graduate School of Medicine, 2-2 Yamadaoka, Suita, Osaka Japan; 5 Department of Orthopaedic Biomaterial Science, Osaka University Graduate School of Medicine, 2-2 Yamadaoka, Suita, Osaka Japan; 6 Department of Rehabilitation Science, Osaka Health Science University, 1-9-27 Kita-ku Tenma, Osaka, Japan

## Abstract

**Background:**

Osteoarthritis (OA) is one of the most common joint diseases in elderly people, however, the underlying mechanism of OA pathogenesis is not completely clear. Periostin, the extracellular protein, has been shown by cDNA array analysis to be highly expressed in OA, but its function is not fully understood. The purpose of this study was to examine the expression and function of periostin in human OA.

**Methods:**

Human cartilage and synovia samples were used for the analysis of periostin expression and function. The human cartilage samples were obtained from the knees of patients undergoing total knee arthroplasty as OA samples and from the femoral bone head of patients with femoral neck fracture as control samples. Quantitative RT-PCR, ELISA, and immunohistochemistry were used for analysis of periostin expression in cartilage and synovia. Human primary chondrocytes isolated from control cartilage were stimulated by periostin, and the alteration of OA related gene expression was examined using quantitative RT-PCR. Immunocytochemistry of p65 was performed for the analysis of nuclear factor kappa B (NFκB) activation.

**Results:**

The periostin mRNA was significantly higher in OA cartilage than in control cartilage. Immunohistochemical analysis of periostin showed that the main positive signal was localized in chondrocytes and their periphery matrix near the erosive area, with less immunoreactivity in deeper zones. There was positive correlation between Mankin score and periostin immunoreactivity. The periostin expression was also detected in the fibrotic cartilage and tissue of subchondral bone. In cultured human chondrocytes, periostin induced the expression of interleukin (IL)-6, IL-8, matrix metalloproteinase (MMP)-1, MMP-3, MMP-13, and nitric oxide synthase-2 (NOS2) in a dose- and time-dependent manner. The activation of NFκB signaling was recognized by the nuclear translocation of p65. Periostin-induced upregulation of these genes was suppressed by NFκB inactivation in chondrocytes.

**Conclusion:**

Periostin was upregulated in OA cartilage, and it may amplify inflammatory events and accelerate OA pathology.

**Electronic supplementary material:**

The online version of this article (doi:10.1186/s12891-015-0682-3) contains supplementary material, which is available to authorized users.

## Background

Osteoarthritis is a leading cause of disability in the elderly and causes pain, stiffness, and loss of function in articulating joints. It is characterized by progressive cartilage erosion, osteophyte formation, subchondral bone formation, and synovial inflammation, which follow alteration in the biomechanical and biochemical properties of the joints [1].

The details of OA pathogenesis are not fully understood, and there are currently no disease-modifying OA drugs available; thus, treatment is limited to symptomatic relief or surgical replacement of the affected joints. To discover novel molecules for therapeutic targets and/or diagnostic markers, many microarray analyses using RNA isolated from OA cartilage [2, 3], subchondral bone [4], and synovia [5] have been reported.

Some array reports have shown that periostin was upregulated in OA tissues. Loeser et al. reported high transcriptional levels and deposition of periostin on the surface and in the matrix of denatured cartilage in a mouse OA model [6]. Zhang et al. reported that periostin mRNA was upregulated in rat OA subchondral bone at an early stage in a surgical OA model [7]. Geyer et al. reported that periostin was upregulated in damaged cartilage relative to intact cartilage within the same joint of patients with OA of the knee, but further analysis was not reported [8].

Periostin was first identified in a mouse osteoblast cell line as a matricellular protein belonging to the fasciclin family. Expression of periostin has been recognized during embryogenesis [9] and in adult connective tissues subjected to mechanical stress [10]. Periostin can crosslink to other extracellular matrix (ECM) proteins, such as collagen I, fibronectin, and tenascin-C; therefore, periostin is expressed in fibrous to solid connective tissues, such as periosteum [11], tendon, periodontal ligaments [12], blood vessels, and heart valves [13]. In fact, periostin-null mice showed defective collagen cross-links and decreased resistance to mechanical stress [14]. In addition, periostin is re-expressed in fibrous tissues formed after injury and recruits mesenchymal cells by interacting with integrin, which is followed by tissue repair [15]. Actually, periostin-deficient mice exhibit delays in repairing and remodeling of injured tissues, such as skin [16], bone fractures [17], and heart tissues, after myocardial infarction [18].

These reports indicate that periostin has crucial roles in tissue repair. However, in some cases, periostin can accelerate pathogenesis of tumors [19, 20], bronchial asthma [21, 22], atopic dermatitis [23, 24], polycystic kidney disease, and other fibrotic diseases [25]. As recently reported, periostin deposition promotes chronic allergic inflammation by activating nuclear factor kappa B (NFκB) signaling [16, 23, 26].

In this study, we examined periostin mRNA/protein expression in human OA tissues and performed in vitro experiments using human chondrocytes to investigate the effects of periostin in OA pathology.

## Methods

### Clinical samples

**Fig. 1:**
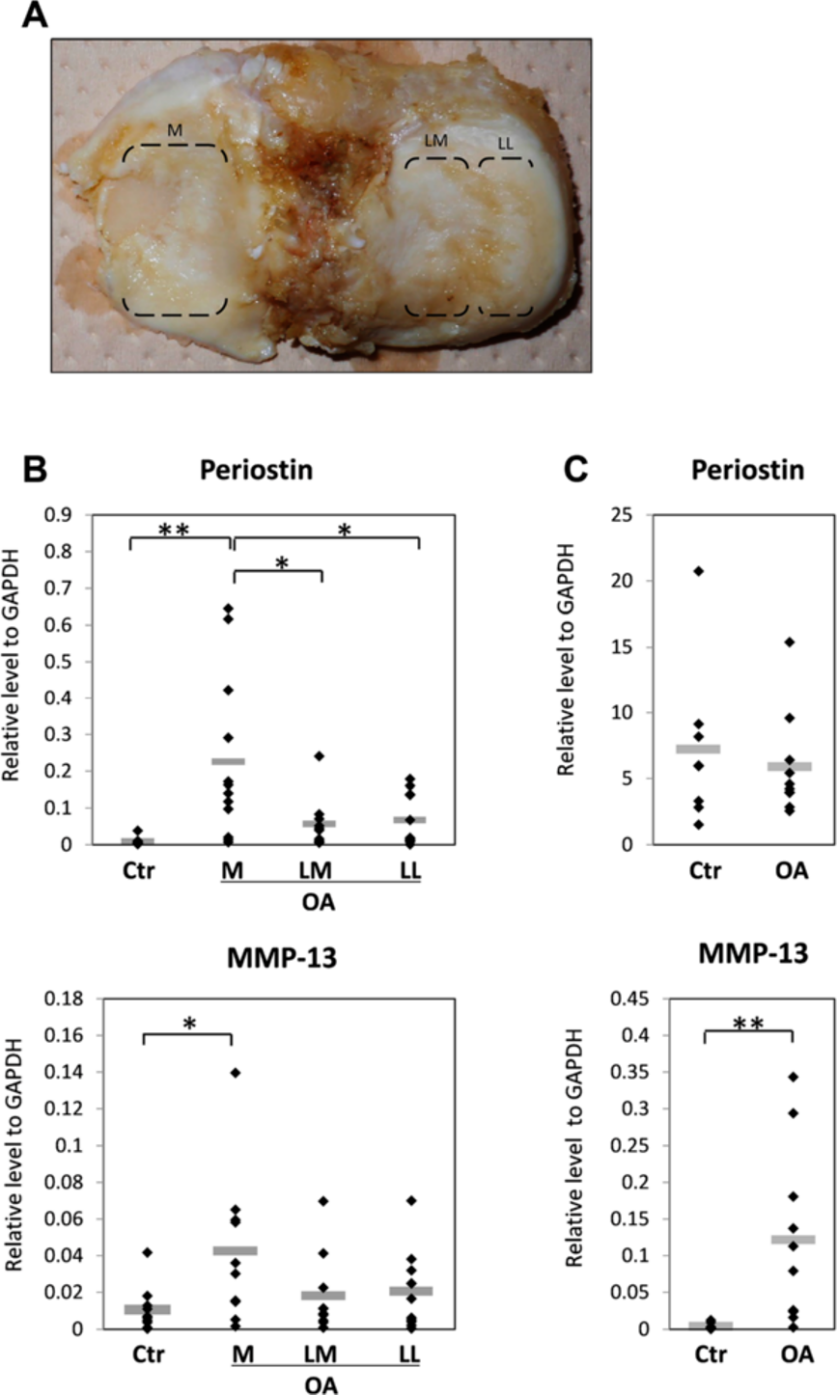
Periostin and MMP-13 gene expression analysis in osteoarthritis (OA). Quantitative RT-PCR analysis was performed using OA and control cartilage or synovial samples. OA cartilage obtained from tibial cartilage of patients who underwent total knee arthroplasty was divided into three parts: M, medial tibial plateau; LM, medial area of the lateral tibial plateau (non-covered area by meniscus); and LL, lateral area of the lateral tibial plateau (covered area by meniscus) (**a**). Control cartilages (Ctr) were obtained from the femoral bone head. Periostin and MMP-13 mRNA expression were significantly high in medial OA cartilage (**b**). However, in synovia, only MMP-13 was significantly upregulated in OA (**c**). Relative expression to GAPDH is shown, and horizontal bars indicate mean values. *; *P* < 0.05. **; *P* < 0.01

This study was approved by the Osaka University Research Ethics Committee and Suita Municipal Hospital Research Ethics Committee, and specimens were taken after patients gave informed consent. Human OA cartilage (*n* = 26; mean ± SD 73.6 ± 8.3 years) (Fig. 1a) and synovial samples (*n* = 10; mean ± SD 72.6 ± 7.5 years) were obtained from patients undergoing total knee replacement surgery for the treatment of clinically diagnosed OA. Control cartilage samples without macroscopic changes were obtained from the femoral bone heads of patients with femoral neck fractures (*n* = 20; mean ± SD 80.1 ± 8.3 years). Control synovia samples were obtained from the knee joints of non-OA, non-rheumatoid arthritis (RA) patients (*n* = 8; age, 28.5 ± 10.1 years) during arthroscopic surgeries.

### Histology and Histochemistry

The OA cartilage tissue samples (*n* = 16) were sagitally cut into 2–4 pieces by bone saw. The specimens were fixed with 10 % formaldehyde solution and decalcified with 10 % ethylenediaminetetraacetic acid (EDTA) (pH 7.4) following delipidation with ethanol. They were embedded in paraffin followed by dehydration with serial ethanol and clearance with xylene. Sagittal 5-μm thick sections were made and stained with toluidine blue for histological grading described by Mankin [27] and used for immunohistochemistry.

Deparaffinized sections were treated with proteinase K (DAKO, California, USA) for 5 min. Endogenous peroxidases were quenched with 3 % hydrogen peroxidase in phosphate-buffered saline (PBS) for 5 min. The sections were blocked with normal rabbit serum (Nichirei, Tokyo, Japan) for 30 min, then incubated in goat anti-human periostin antibody (1:100, Cat. sc-49480; Santa Cruz Biotechnology, Texas, USA) or goat normal IgG (1:100, Cat. sc-2028; Santa Cruz Biotechnology) for 1 h. After the reaction with peroxidase-conjugated anti-goat IgG secondary antibody (Nichirei) for 30 min, positive signal color developed with diaminobenzidine solution (Nichirei) for several minutes. The slides were counterstained with hematoxylin, dehydrated, and enclosed with Entellan New (Merck, Darmstadt, Germany). All procedures were performed at room temperature. During each step, the slides were washed with 0.1 % tween-20 including PBS for three times.

### Immunohistochemical (IHC) Scoring

**Fig. 2:**
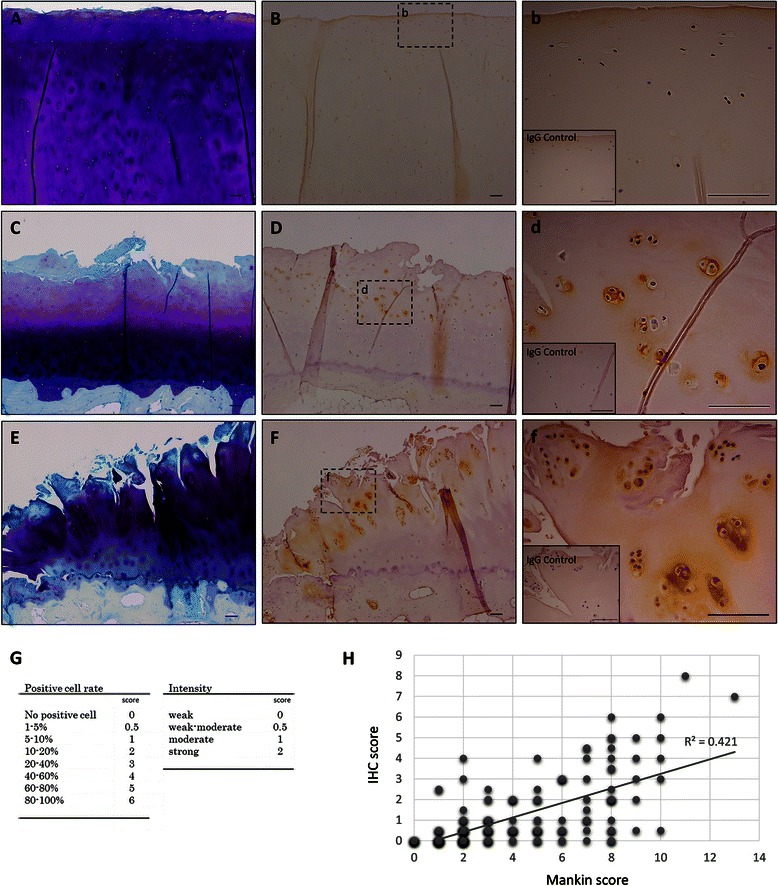
Periostin localization in human OA cartilage. Toluidine blue staining (**a**, **c**, **e**) and periostin immunostaining (**b**, **d**, **f**, *b*, *d*, *f*) of human osteoarthritis (OA) cartilage. Right panels (*b*, *d*, *f*) are high magnification images of the middle panels (**b**, **d**, **f**), respectively. The inset in the right panels shows the isotype control. The different OA grade samples are shown; near the intact cartilage (**a**, **b**, *b*) (Mankin score, 1; Positive cell rate, 0; Intensity, 0), moderate OA cartilage (**c**, **d**, *d*) (Mankin score, 8; Positive cell rate, 4; Intensity 1), and severe OA cartilage (**e**, **f**, *f*) (Mankin score, 11; Positive cell rate, 6; Intensity 2). In denatured cartilage, periostin was localized in chondrocytes and their periphery matrix particularly near the erosive surface. Bars = 100 μm. Positive correlation between IHC score (**g**) and Mankin score was observed (**h**). (r = 0.649, *P* < 0.001)

For evaluation of the periostin expression in OA cartilage, the stained sections were graded on positive cell rate and intensity of immunostaining by two authors concurrently (Fig. 2g) [28, 29]. The percentage of periostin positive cells was estimated and assigned to 8 categories: 0 % (0), 1–5 % (0.5), 5–10 % (1), 10–20 % (2), 20–40 % (3), 40–60 % (4), 60–80 % (5), and > 80 % (6). The intensity of immunostaining was scored as the following: weak (0), weak–moderate (0.5), moderate (1), and strong (2). Three to five snapshots of the specimens (about 3-mm square) were randomly taken per one specimen.

### Cell culture and stimulation with periostin

For the in vitro assay, we used human chondrocytes derived from femoral bone head. Cartilage samples were minced with a scalpel and digested with 0.3 U/mL collagenase NB4 (SERVA Electrophoresis, Land Baden-Württemberg, Germany)–Dulbecco’s Modified Eagle Medium (DMEM) supplemented with 10 % fetal bovine serum (FBS) and incubated overnight at 37 °C. The suspension was passed through 40-μm pore filters, and then the cells were washed three times with PBS and fed in culture dishes with DMEM supplemented with 10 % FBS, 1 % antibiotic antimycotic (Sigma–Aldrich, Saint Louis, USA) and incubated at 37 °C with 5 % humidified CO_2_. Within 3 days, only the adhered cells were collected by trypsin/EDTA, and replated at 3 × 10E4 cells/well in 96-well plates, and incubated overnight. The cells were treated with human recombinant periostin (0, 3, 10, and 30 μg/mL) (R&D Systems, Minnesota, USA) and/or BAY11-7082 (0, 1, and 5 μM) (Sigma–Aldrich) in DMEM supplemented with 10 % FBS for an appropriate time (1, 3, 6, 12, 24, and 72 h). All in vitro experiments were treated with the same volume vehicle; PBS for periostin and dimethylsulfoxide (DMSO) for BAY11-7082. Cells were used for RNA extraction and the culture supernatants of chondrocytes were preserved at −20 °C until use in ELISA assay.

### RNA extraction, cDNA synthesis, quantitative reverse transcription polymerase chain reaction (RT-PCR)

OA cartilage tissue from the joint surface of the tibia was first divided into three parts: the medial plateau, meniscus covering area of the lateral plateau, and meniscus-uncovered lateral plateau (Fig. 1a). Prior to total RNA extraction, divided cartilage tissues were minced and digested with 0.3 U/mL collagenase NB4 in 10 % FBS-DMEM and incubated overnight at 37 °C. After washing with PBS three times, collected cells were used for total RNA extraction.

Synovial tissues were thoroughly minced and preserved at −80 °C, after which extraction of total RNA from the clinical sample was performed using an RNeasy fibrous kit (QIAGEN, Hilden, Netherland) according to the manufacturer’s protocol.

The extraction of total RNA from human cultured chondrocytes was performed using NucleoSpin XS (Takara, Shiga, Japan) according to the manufacturer’s protocol.

For quantitative RT-PCR, total RNA was reverse transcribed into first-strand complementary DNA (cDNA) using Super Script VILO (Life Technologies, Maryland, USA) and random primers. The PCR amplification was performed using Fast SYBR green master mix (Life Technologies) and StepOne Plus (Life Technologies). Target transcriptional levels were normalized to the level of glyceraldehyde 3-phosphate dehydrogenase (GAPDH) expression. The primers are listed in Additional file 1.

### Enzyme-linked immunosorbent assay (ELISA)

Human matrix metalloproteinase (MMP)-13 and interleukin (IL)-6 in culture supernatant were measured using ELISA kits (RayBiotech, Georgia, USA). Protocols, range, sensitivity, and interassay precisions were as described by the manufacturer’s technical sheets.

### Immunocytochemistry

For immunostaining, human primary chondrocytes were seeded on chamber slides (Thermo Scientific, Massachusetts, USA), and stimulated with 20 μg/mL periostin for 3 h. The cells were fixed with 4 % paraformaldehyde for 10 min, permeabilized with 0.1 % Triton X-100 in PBS for 15 min, and treated with 1 % bovine serum albumin-PBS for 30 min. The cells were incubated with rabbit anti-p65 antibody (1:100, Cat. sc109; Santa Cruz Biotechnology) for 1 h and then with Alexa Fluor 488-conjugated anti-rabbit secondary antibody (1:300, Thermo Scientific) for 1 h. Nuclei were stained with DAPI. All procedures were performed at room temperature. Between steps, the cells were washed three times with 0.1 % tween-20 including PBS.

### Statistical analysis

The results of in vitro mRNA analysis are presented as means ± RQ Max/Min values and other results are presented as means ± SD. All in vitro experiments were performed using samples from at least three donors. Student’s unpaired *t*-test was used to perform statistical analysis of experimental data, with Bonferroni correction as needed. Pearson correlation analysis was used for the analysis of the relationships between parameters. *P* values less than 0.05 were considered significant.

## Results

### Expression of periostin mRNA in OA cartilage and synovia

The relative mRNA expression levels of periostin and MMP-13 (target/GAPDH ratio) in clinical samples was measured by quantitative RT-PCR. Since OA tibial cartilages (*n* = 10; mean ± SD 75.8 ± 7.3 years) vary in denaturing degree by a part, it was divided into three parts: medial tibial plateau, medial area of the lateral tibial plateau, and lateral area of the lateral tibial plateau. The cartilage of femoral bone heads obtained from patients with femoral neck fractures was used as control (*n* = 10; mean ± SD 80.8 ± 5.2 years). The expression levels of periostin in cartilages were significantly higher in medial tibial plateaus (mean ± SD 0.226 ± 0.22) than in the lateral tibial plateaus (mean ± SD 0.0631 ± 0.069) and in control cartilages (mean ± SD 0.00948 ± 0.01). In addition, MMP-13 expression levels were significantly higher in the medial tibial plateaus (mean ± SD 0.0426 ± 0.04) than those in control cartilages (mean ± SD 0.0107 ± 0.012) (Fig. 1b). On the other hand, in synovia, there were no significant difference in periostin expression between OA (*n* = 10; mean ± SD 72.6 ± 7.5 years, 5.906 ± 3.89) and control (*n* = 8; mean ± SD 28.5 ± 10.1 years, mean ± SD 7.215 ± 6.06), although MMP-13 was significantly upregulated in OA synovia (mean ± SD 0.122 ± 0.12) in comparison with control synovia (mean ± SD 0.00432 ± 0.0044). (Fig. 1c).

### Immunohistochemical assay of periostin in OA cartilage

To histologically confirm periostin localization in OA tissues, we attempted to detect periostin in OA cartilages (*n* = 16; mean ± SD 72.0 ± 8.8 years) with a specific antibody. In the intact area of cartilages indicated by uniform toluidine blue staining, the positive staining of periostin was scarcely detected (Fig. 2a, b, *b*). The mild-to-moderate OA cartilage areas were characterized by the wearing of surface and some cleft indicated by less toluidine blue staining (Fig. 2c). Positive staining of periostin was detected in chondrocytes and lacuna located near the erosive area (Fig. 2d, *d*). The severe OA cartilage areas lost majority of their matrix and had many deep clefts (Fig. 2e). Periostin expression was also detected in many chondrocytes and their peripheral matrix in the erosive surface (Fig. 2f, *f*). However, in the deeper zone of cartilage, the positive staining was rarely detected in chondrocytes and matrices (Fig. 2d, f).

To evaluate the correlation of periostin expression and histological grading, we scored the OA pathophysiology and periostin immunoreactivity with Mankin score and IHC scoring (Fig. 2g), respectively. IHC score was settled in consideration positive of cell rate and immunointensity. As shown in Fig. 2h, periostin expression levels were positively correlated with cartilage degeneration (*r* = 0.649, *P* < 0.001).

**Fig. 3:**
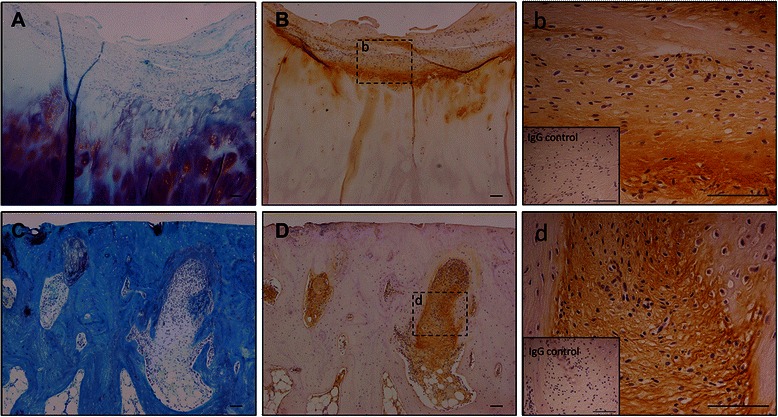
Periostin localization in OA fibrotic tissues. Toluidine blue staining (**a**, **c**) and periostin immunostaining (**b**, **d**, *b*, *d*) of human osteoarthritis cartilage and subchondral bone. Right panels (*b*, *d*) are high magnification images of the middle panels (**b**, **d**), respectively. The inset in the right panels shows the isotype control. Periostin localized fibrotic area on cartilage surface (**a**, **b**, *b*) and in subchondral bone of full-thickness cartilage defect (**c**, **d**, *d*). Bars = 100 μm

Besides, positive staining of periostin was also detected in fibrous tissue on the cartilage surface, which was estimated to be derived from synovial tissue or fibrotic denaturing cartilage (Fig. 3a, b, *b*). In the full-thickness cartilage defect area, fibrosis of subchondral marrow was observed, and periostin was expressed in these fibrotic areas (Fig. 3c, d, *d*).

### The effects of periostin on OA-related gene expression in human chondrocytes

**Fig. 4:**
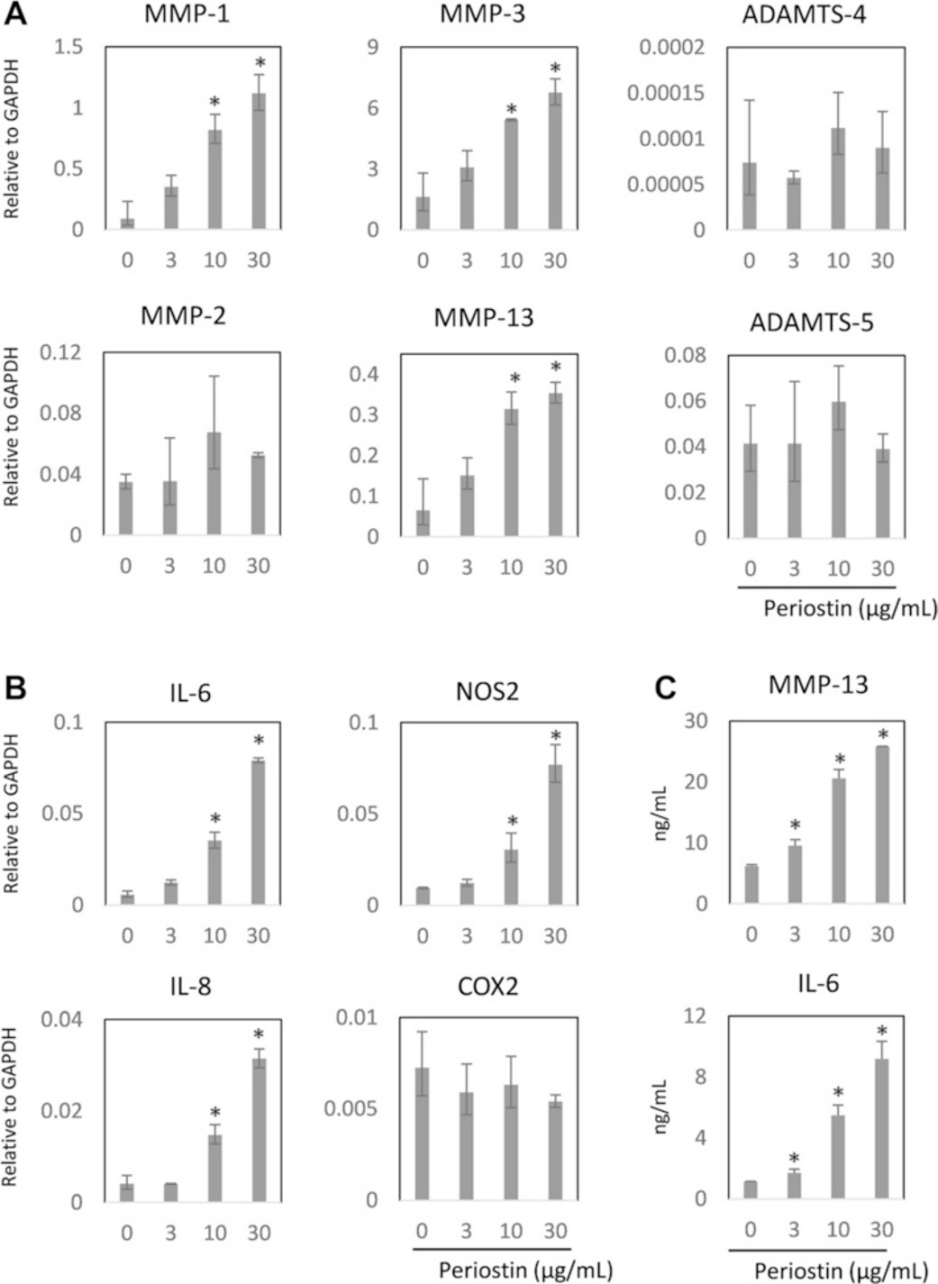
The effect of Periostin for the expression of catabolic enzymes and inflammatory cytokines in human cultured chondrocytes. Human primary chondrocytes were cultured in media supplemented with periostin (0, 3, 10, and 30 μg/mL) for 24 h. Alteration of osteoarthritis (OA)-related gene expression was assessed by quantitative RT-PCR for cultured chondrocytes (**a**, **b**) and ELISA for the culture supernatant (**c**). The upregulation of osteoarthritis (OA)-related catabolic enzymes MMP-1, −3, and −13 were confirmed by quantitative RT-PCR for cultured chondrocytes, but MMP-2, ADAMTS-4, and −5 were not affected by periostin (**a**). Inflammatory cytokines, IL-6, −8, and NOS2 were also upregulated by periostin in chondrocytes, but COX-2 was not affected by periostin stimulation (**b**). The target mRNA levels are shown relative to GAPDH. MMP-13 and IL-6 protein production were also compatibly upregulated in culture supernatant of chondrocytes stimulated by periostin (**c**). Representative data from one of three donors are shown. *, *P* < 0.05

**Fig. 5:**
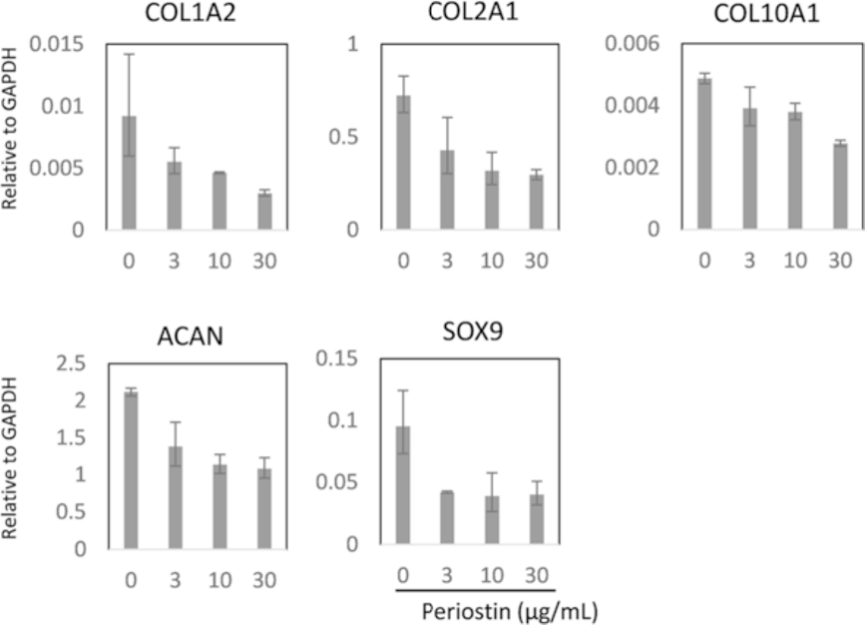
The effect of periostin for the expression of chondrocytic genes in human cultured chondrocytes. The alteration of chondrocytic genes expression was assessed by quantitative RT-PCR for cultured chondrocytes after stimulation by periostin in variant doses (0, 3, 10, 30 μg/mL). The expressions of COL1A2, COL2A1, COL10A1, ACAN, and SOX9 gene were unaffected by periostin. The target mRNA levels are shown relative to GAPDH. Representative data from one of three donors are shown. *, *P* < 0.05

To assess the effects of periostin on OA-related gene expression, human primary chondrocytes isolated from the control cartilage were stimulated by various concentrations of periostin (0, 3, 10, 30 μg/mL) for 24 h. We repeated the same experiment with chondrocytes derived from three different donors. First, as showed in Fig. 4a, we investigated the expression of catabolic enzymes related to OA, specifically MMP-1, MMP-2, MMP-3, MMP-8, MMP-9, MMP-13, a disintegrin and metalloproteinase with thrombospondin motifs (ADAMTS)-4, and ADAMTS-5. The expressions of MMP-1, MMP-3, and MMP-13 were significantly upregulated dose-dependently, but the expression of MMP-2 was not affected by periostin stimulation. MMP-8 and MMP-9 were not detected in cultured chondrocytes (data not shown). The results of ADAMTS-4 and ADAMTS-5 analysis were not consistent among all donors. Second, we examined the alteration of inflammatory and other gene mRNA levels, including IL-1, IL-6, IL-8, tumor necrosis factor (TNF) α, cyclooxygenase-2 (COX-2), and nitric oxide synthase-2 (NOS2) (Fig. 4b). Expressions of IL-1 and TNF α were induced in the periostin high-dose group but near the detection threshold (data not shown). The expressions of IL-6, IL-8, and NOS2 were remarkably upregulated in a dose-dependent manner, but the expression of COX-2 was not altered by periostin stimulation. For protein level, the same trend was observed for MMP-13 and IL-6 by performing an ELISA assay in culture supernatants (Fig. 4c). Finally, we confirmed the alteration of chondrocytic gene expression, specifically Collagen type (COL) 1, 2, 10, Aggrecan (ACAN), and SRY-related HMG box (SOX9). However, these genes were not consistently affected by periostin stimulation (Fig. 5).

The endogenous mRNA levels of these catabolic, inflammatory, anabolic genes were different among donors; however, same trend of periostin effect was observed in all donors. (see Additional file 2).

### Time course analysis of OA-related gene expression in periostin-stimulated chondrocytes

**Fig. 6:**
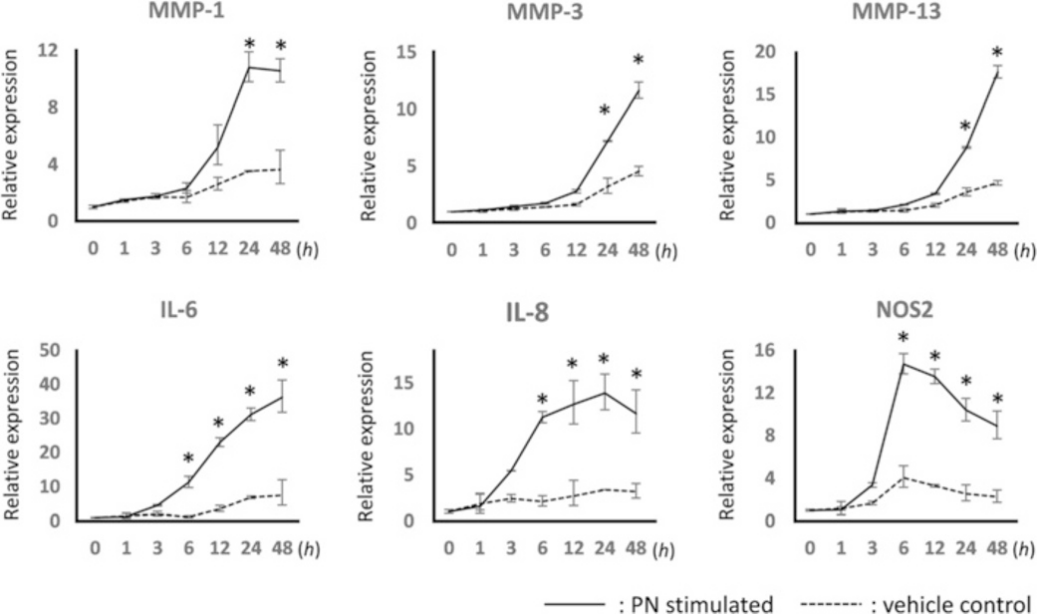
Periostin upregulated MMP-1, MMP-3, MMP-13, IL-6, IL-8, and NOS2 in a time-dependent manner. Human primary chondrocytes were stimulated by 20 μg/mL periostin, and the mRNA levels were measured by quantitative RT-PCR at different time points. The solid lines and broken lines represent the periostin treated group and control group respectively. The relative expression compared with at time zero is shown. Representative data from one of three donors are shown.*, *P* < 0.05

The transcription levels of MMP-1, MMP-3, MMP-13, IL-6, IL-8, and NOS2 genes were measured at different time points after addition of 20 μg/mL periostin by quantitative RT-PCR. The mRNA levels of IL-6, IL-8, and NOS2 were significantly higher after 6 h and the mRNA levels of MMP-1, MMP-3, and MMP-13 were higher after 24 h in periostin-stimulated chondrocytes than that in PBS treated chondrocytes. The IL-8 mRNA level remained stable after 6 h but NOS2 mRNA level gradually decreased after 6 h. However, the levels of other genes continued to increase until the assay endpoint (48 h) (Fig. 6).

### Periostin act on chondrocytes via NFκB signaling

**Fig. 7:**
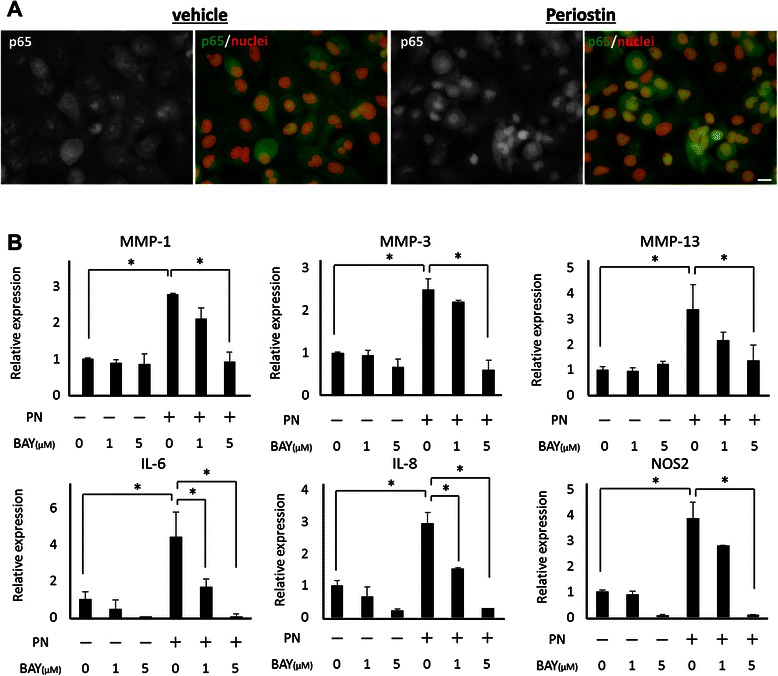
Periostin-induced upregulation of catabolic enzymes and inflammatory cytokines via the NF-κB signaling. (**a**) Human primary chondrocytes were stimulated by 20 μg/mL periostin, and the nuclear translocation of p65 was determined by immunocytochemistry at 3 h. Gray and green represent p65. Red represent nucleus. Bar = 10 μm. (**b**) Human primary chondrocytes were exposed to 20 μg/mL periostin and/or different concentrations of BAY11-7082 for 24 h. The mRNA levels of IL-6, IL-8, MMP-1, MMP-3, MMP-13, and NOS2 were measured by quantitative RT-PCR. The relative expression compared to vehicle control is shown. Representative data from one of three donors are shown. *, *P* < 0.05

From the fact that inflammatory cytokines were upregulated by periostin, we investigated the relationship between NFκB signaling and periostin in chondrocytes. As shown in Fig. 7a, p65 which is a component of NFκB signaling was detected in chondrocyte by immunostaining. Although, the nuclear location of p65 was recognized in chondrocytes of vehicle control, periostin stimulated chondrocytes exhibited more strong signal in their nucleus. Furthermore, BAY11-7082, an inhibitor of NFκB signaling by suppressing IκB phosphorylation, suppressed the periostin-induced expression of MMP-1, MMP-3, MMP-13, IL-6, IL-8, and NOS2 in a dose-dependent manner (Fig. 7b).

## Discussion

The main objective of our study was to elucidate the expression and function of periostin in human OA tissue. First, we verified the upregulation of periostin mRNA level in OA tissues using isolated chondrocytes and synovia. Because arthritic progression differs depending on the cartilage area, OA tibial cartilage was separated into three parts. In the medial OA, the cartilage of the medial tibial plateau was the most denatured area by mechanical pressure. The cartilage of the lateral tibial plateau showed less damage than the medial side, particularly in the area covered by the lateral meniscus [30]. To obtain control cartilage, we used the cartilage of the femoral bone head obtained from patients who underwent bipolar hip arthroplasty; this cartilage showed little degeneration regardless of age and is used as control in many studies [31–33]. The medial tibial plateau had the highest periostin mRNA level compared with that in the femoral bone head and other areas in OA cartilage. In the present study, we treated the cartilages with collagenase prior to the extraction of total RNA, because it is difficult to extract the total RNA from hard and ECM-rich tissues such as bone and cartilage [8, 34, 35]. Although isolation stress by the enzymatic treatment of cartilage might have some effect on the mRNA profile, the proportion of MMP-13 expression depended on the degeneration of cartilage and was consistent with the expressions in previous reports [36]. Both control and OA synovia showed the high expression of periostin, whereas MMP-13 was significantly increased in OA synovia compared with that in control synovia. Because many fibrous tissues express periostin [10, 15], it is probable that synovia also expresses periostin constantly.

Although mRNA expression of periostin is known to occur in OA chondrocytes [6, 8], the localization of periostin remains unclear. In our results, the positive staining of periostin was detected in chondrocytes and their periphery matrix in the erosive surface layer, particularly near the clefts of denaturing cartilage. It is believed that mechanical stress is the major etiology in OA [37, 38]. The surface layer of cartilage is exposed to higher pressure and shear stress than the other layers, which cause scuffing and cracks. In the deeper zone, periostin was rarely detected; nevertheless, surface cartilage was strongly denatured. Perhaps, periostin may possibly be upregulated in chondrocytes in response to mechanical stress. In fact, periostin was detected in the many connective tissues resistant to mechanical loading, such as skin, tendons, and ligaments [10]. Moreover, some cells expressed periostin highly in response to mechanical stress and/or tension [39–41]. However, it is difficult to imitate in vivo stress condition in ex vivo and/or in vitro study; therefore, we could not elucidate what induces periostin expression in OA.

To investigate the effect of periostin, we verified the alteration of OA-related markers in cultured chondrocytes by stimulation with periostin [19]. Our results showed that periostin induced the expression of MMP-1, MMP-3, and MMP-13 in chondrocytes, and they are known to be related in OA pathogenesis [42, 43]. Some studies have reported the relationship between periostin and MMPs in several cell types. Periodontal ligament cells expressed MMP-2 by periostin stimulation through the integrin/ERK pathway [44]. Periostin also induces the secretion of MMP-2 and MMP-13 from vascular endothelial cells, MMP-2 from epithelial cells, and MMP-9 from bone marrow macrophages [45]. Different types of MMPs are induced by periostin in various types of cells, which suggest that there are different pathways depending on cell type. These MMPs may have a role in remodeling/repair of injured fibrotic tissues, however, in cartilage, MMP-1, MMP-3, and MMP-13 are thought to be the crucial collagenases in OA and exhibit increased expressions in human OA cartilage [46]. Moreover, in our study, periostin did not upregulate collagen genes such as COL1 and COL2; it is believed that periostin may accelerate ECM destruction in OA without synthesizing new matrices.

In addition to MMPs, IL-6 and IL-8 were upregulated by periostin stimulation in chondrocytes, which suggests that there is a relationship between periostin and inflammatory events in OA. Inflammatory cytokines, such as IL-1, IL-6, IL-8, and TNFα have a role in cartilage degradation [47]. Many events in OA pathological processes are thought to be mediated by inflammatory cytokines. In fact, these cytokines stimulate the expression of MMPs in chondrocytes and synoviocytes [48, 49]. In our time course analysis, periostin induced inflammatory cytokines earlier, followed by the upregulation of MMPs, which indicate that periostin induced the expression of MMPs through inflammatory cytokines in chondrocytes. Some studies have suggested that periostin deposition causes chronic inflammation [50, 22], and NFκB signaling has been proven to be downstream signaling induced by periostin [16, 23, 26]. Our results showed that periostin precipitated the nuclei translocation of p65 in human cultured chondrocytes, and NFκB inhibitor suppressed the periostin-induced upregulation of not only inflammatory cytokines but also NOS2, MMP-1, MMP-3, and MMP-13. NOS2 and MMPs are already known to be induced by inflammatory cytokines through NFκB signaling in human chondrocytes [48, 51]. It is suggested that in human chondrocytes, periostin activates NFκB signaling, followed by the upregulation of inflammatory cytokines and MMPs.

Some limitations exist in this study. First, because we used chondrocytes derived from femoral bone head without dedifferentiation in vitro, they may have different phenotypes from OA chondrocytes. Second, because it is difficult to estimate how much periostin is deposited in cartilage matrix or how much periostin affects the chondrocytes in vivo, our in vitro study may not imitate the in vivo microenvironment. Accordingly, further experiments on how periostin acts in in vivo conditions are required to confirm the causal relationship to OA pathogenesis.

## Conclusion

This report demonstrated periostin expression in human OA and the effects of periostin in human primary chondrocytes. Periostin was detected in chondrocytes and their peripheral matrices in degraded cartilage. In our in vitro study, primary chondrocytes expressed the inflammatory cytokines and MMPs in response to periostin. Periostin may accelerate the pathogenesis of OA.

## Additional files

Additional file 1: **Primer list for qRT-PCR.** (XLSX 10 kb) Additional file 2: **Periostin upregulated OA related genes in a similar trend among all donors.** The same trend of periostin effects was observed in two other donors by qRT-PCR for cells (A) and ELISA for culture supernatant (B), although the endogenous expression of OA related catabolic, inflammatory, anabolic genes was different among donors. *, *P* < 0.05. (PDF 145 kb)

## Abbreviations

OA: Osteoarthritis
RT-PCR: Reverse transcription polymerase chain reaction
ELISA: Enzyme-Linked Immunosorbent Assay
IL-1: Interleukin 1
IL-6: Interleukin 6
IL-8: Interleukin 8
TNFα: Tumor necrosis factor α
MMP-1: Matrix metalloproteinase 1
MMP-3: Matrix metalloproteinase 3
MMP-8: Matrix metalloproteinase 8
MMP-9: Matrix metalloproteinase 9
MMP-13: Matrix metalloproteinase 13
NOS2: Nitric oxide synthase-2
NFκB: Nuclear factor kappa B
RA: Rheumatoid arthritis
EDTA: Ethylenediaminetetraacetic acid
PBS: Phosphate-buffered saline
DMEM: Dulbecco’s Modified Eagle Medium
DMSO: Dimethylsulfoxide
GAPDH: Glyceraldehyde 3-phosphate dehydrogenase
ADAMTS-4: A disintegrin and metalloproteinase with thrombospondin motifs-4
ADAMTS-5: A disintegrin and metalloproteinase with thrombospondin motifs-5
COX-2: Cyclooxygenase-2
COL 1: Collagen type 1
COL 2: Collagen type 2
COL 10: Collagen type 10
ACAN: Aggrecan
SOX9: SRY-related HMG box 9
