# Supplementary figures and images for: Expression and pathological effects of periostin in human osteoarthritis cartilage
Source: BMC Musculoskelet Disord. 2015 Aug 21;16:215. doi: 10.1186/s12891-015-0682-3 (PMC4545863; doi:10.1186/s12891-015-0682-3)

**A**

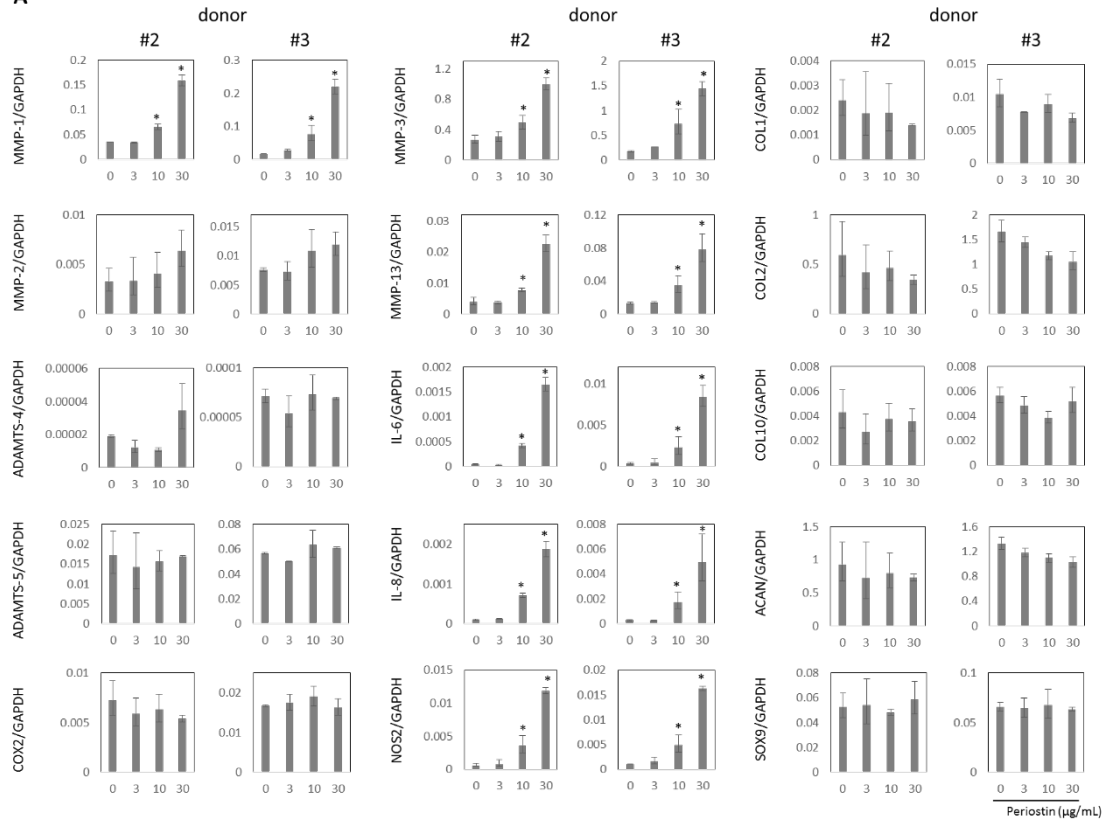

**B**

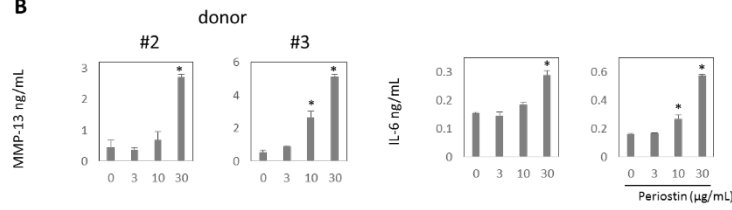

Supplement: Additional file 2: — Periostin upregulated OA related genes in a similar trend among all donors. The same trend of periostin effects was observed in two other donors by qRT-PCR for cells (A) and ELISA for culture supernatant (B), although the endogenous expression of OA related catabolic, inflammatory, anabolic genes was different among donors. *, P < 0.05. (PDF 145 kb) [file 12891_2015_682_MOESM2_ESM.pdf]
